# Editorial for the Special Issue on Micro-Electromechanical System Inertial Devices

**DOI:** 10.3390/mi14122134

**Published:** 2023-11-21

**Authors:** Huiliang Cao

**Affiliations:** State Key Laboratory of Dynamic Measurement Technology, North University of China, Taiyuan 030051, China; caohuiliang@nuc.edu.cn

Micro-electromechanical systems (MEMS) are miniature systems comprising micro-mechanical sensors, actuators, and microelectronic circuits [1]. With the explosive growth of core technologies such as MEMS miniaturization, microelectronic integration, and high-precision mass production [2], various types of MEMS sensors have found wide-ranging applications in fields such as aerospace [3], petrochemicals [4], the marine and automotive industries [5], household appliances [6], and healthcare [7]. In this context, MEMS inertial devices represent a core category of MEMS sensors. They are designed to capture physical motion, such as linear displacement or angular rotation, and convert these responses into electrical signals, which are subsequently amplified and processed through electronic circuits [8]. MEMS inertial devices primarily include MEMS gyroscopes [9], MEMS accelerometers [10], MEMS magnetometers [11], and MEMS-IMUs [12]. Accelerometers and gyroscopes are the most common MEMS inertial sensors. The former are sensitive to axial acceleration and convert it into usable output signals [13], while gyroscopes detect the angular velocity of a moving body relative to inertial space [14]. Furthermore, the combination of three MEMS accelerometers and three MEMS gyroscopes forms a micro-inertial measurement unit (MIMU) capable of sensing linear acceleration in three directions and angular acceleration in three directions [15]. In summary, MEMS inertial microsystems employ three-dimensional heterogeneous integration technology, integrating MEMS accelerometers, gyroscopes, pressure sensors, magnetic sensors, signal processing circuits, and embedded algorithms into silicon chips to achieve chip-level guidance, navigation, positioning, and other functionalities [16].

In terms of applications, MEMS inertial devices are mainly used to provide accurate position and motion measurement solutions for aerospace, underwater exploration, robot control and many other core fields. Take NASA’s Mars rover as an example, where MEMS accelerometers and gyroscopes enable the rover to land and navigate precisely on the Martian surface. These sensors are, therefore, essential for space exploration and can ensure accurate data collection and telemetry [17]. In the field of underwater exploration, MEMS inertial sensors play an important role in navigation for autonomous underwater vehicles in complex underwater terrain. The REMUS AUV, developed by Woods Hole Oceanographic Institution, uses MEMS inertial sensors to map the seabed and conduct scientific research [18]. The integration of MEMS inertial sensors into robots has enhanced the capabilities of robots in industrial automation. Boston Dynamics’ four-legged robot Spot employs MEMS sensors for balance and stability, allowing it to traverse uneven terrain with amazing agility and precision [19]. In the field of automatic driving, MEMS inertial sensors are an indispensable component for safe and efficient navigation. Tesla and other companies use MEMS-based inertial measurement units (IMUs) to provide accurate real-time data for their automatic driving system, ensuring accurate control of vehicle movement and improving the safety and reliability of automated driving technology [20]. The emergence of wearable devices has brought MEMS inertial sensors closer to our daily lives. Fitness trackers such as Fitbit utilize MEMS accelerometers to monitor physical activity and track steps taken by the user, providing valuable health insights [21].

On the other hand, MEMS inertial devices also face a number of challenges, such as low measurement accuracy due to temperature, noise, and their own limitations. They are deployed in extremely variable working environments, facing high temperatures, high pressures, high inertia levels, and high impacts, resulting in demanding requirements for the stability and adaptability of MEMS inertial devices [22]. In order to fully develop and utilize the potential of these devices for high-end manufacturing and cutting-edge applications, researchers and engineers must study their structural optimization, measurement and control systems, manufacturing technologies, and integration applications. This Special Issue presents the latest advances in MEMS inertial devices with the purpose of encouraging readers to explore each article.

This Special Issue contains 11 papers covering different inertial devices such as MEMS accelerometers (Contributions 1–5), MEMS gyroscopes (Contributions 6–10), and inertial navigation systems (Contribution 11). Half of these papers discuss the design and manufacture of inertial devices, such as anchor stress relief (Contribution 1), modal simulation, and structural optimization (Contributions 3 and 4), closed-loop drive circuit design (Contribution 7), and the development of pattern-matching closed-loop systems (Contribution 10). The other half of the papers focus on the optimization and maintenance of MEMS inertial devices, including noise removal (Contribution 2), hardware compensation (Contribution 5), algorithm compensation (Contribution 6), and fault diagnosis (Contribution 8). Additionally, the effects of the resonator’s coupling efficiency on the scale factor of the fiber resonator gyroscope is discussed in (Contribution 9), providing a theoretical reference and experimental basis for various applications on land, sea, and air. The accuracy of inertial units in navigation and positioning systems is discussed in (Contribution 11).

In particular, Liu et al. (Contribution 1) improved the performance of an all-silicon accelerometer by adjusting the ratio of the Si-SiO_2_ bonding area and the Au-Si bonding area in the anchor zone to eliminate the stress. Their results show that the zero-bias full-temperature stability and scale-factor full-temperature stability can be improved significantly. Cui et al. (Contribution 3) manufactured a three-pole-plate dual-capacitance acceleration sensor and used COMSOL, ANSYS and other software to simulate and optimize its structure and shock characteristics. They obtained a sensor with self-powered output, a high output voltage amplitude, and low spurious interference, which can reliably receive vibration signals. Shi et al. (Contribution 4) proposed a piezoelectric MEMS accelerometer (PMA) with four cantilever beams integrated with inertial mass elements to meet the requirement of high-sensitivity acceleration for vector hydrophones. They established a theoretical model for energy harvesting for a piezoelectric cantilever beam, and the geometric size and structure of their micro-device are optimized to meet the vibration absorption conditions. Han et al. (Contribution 7) analyzed the problem of automatic gain control driving a low-Q micromachined gyroscope at room temperature and atmospheric pressure. They proposed a drive circuit based on frequency modulation, which uses the second harmonic demodulation circuit to eliminate the same frequency coupling between the drive signal and the displacement signal. Wu et al. (Contribution 10) proposed a VCF-based mode-matching micromachine-optimized tuning fork gyroscope, which can maximize the scale factor and avoid the use of additional orthogonal rings. On this basis they established a mode-matching, closed-loop system without quadrature-nulling loop, and the corresponding convergence and matching error were quantitatively analyzed.

In the optimization and maintenance of inertial devices, Wang et al. (Contribution 2) proposed a hybrid algorithm based on empirical mode decomposition (EMD) and time–frequency peak filtering (TFPF) to deal with the noise during accelerometer calibration. Faced with the problem of temperature drift, Liu et al. (Contribution 5) designed a combined compensation method using reference voltage source compensation and accelerometer terminal temperature compensation, based on the idea of hardware compensation. They comprehensively improved the performance of the accelerometer within a wide temperature range. Li et al. (Contribution 6) adopted the idea of algorithmic compensation and combined empirical mode decomposition (EMD), a radial basis function neural network (RBF NN), a genetic algorithm (GA) and the Kalman filter (KF) to propose a new fusion algorithm to remove the influence of environment and accurately compensate the temperature drift of a MEMS gyroscope. In order to achieve reliable maintenance, Cui et al. (Contribution 8) designed a dual-mass MEMS gyroscope fault diagnosis platform, which integrates the Simulink structure model of the gyroscope and the measurement and control system, and reserves a variety of algorithm interfaces for users to independently program. This can effectively identify and classify the seven signals of the gyroscope: normal, bias, blocking, drift, multiplicity, cycle, and internal fault. On the applications side, Li et al. (Contribution 9) evaluated the effect of the coupling efficiency of fiber ring resonators on scale factors. This provides a theoretical reference and experimental basis for various applications at sea, on land, and in space. In order to improve the positioning accuracy of shearers, Lu et al. (Contribution 11) established an experimental ground shearer installation based on the positioning model of a tri-INS and discussed the influence of inertial navigation system installation parameters on positioning accuracy.

I would like to take this opportunity to thank all the authors for submitting their papers for this special issue. I would also like to thank all the reviewers for dedicating their time and helping to improve the quality of the submitted papers.
